# Cerebrospinal fluid real‐time quaking‐induced conversion is a robust and reliable test for sporadic creutzfeldt–jakob disease: An international study

**DOI:** 10.1002/ana.24679

**Published:** 2016-06-01

**Authors:** Lynne I. McGuire, Anna Poleggi, Ilaria Poggiolini, Silvia Suardi, Katarina Grznarova, Song Shi, Bart de Vil, Shannon Sarros, Katsuya Satoh, Keding Cheng, Maria Cramm, Graham Fairfoul, Matthias Schmitz, Inga Zerr, Patrick Cras, Michele Equestre, Fabrizio Tagliavini, Ryuichiro Atarashi, David Knox, Steven Collins, Stéphane Haïk, Piero Parchi, Maurizio Pocchiari, Alison Green

**Affiliations:** ^1^ National CJD Research & Surveillance Unit, Western General HospitalUniversity of EdinburghEdinburghScotlandUnited Kingdom; ^2^ Department of Neurological SciencesNational Institute of HealthRomeItaly; ^3^ Institute of Neurological SciencesScientific Institute for Research, Hospitalization and Health CareBolognaItaly; ^4^ Department of Neurodegenerative DiseasesScientific Institute for Research, Hospitalization and Health Care, Carlo Besta Neurological InstituteMilanItaly; ^5^ Sorbonne UniversitiesPierre and Marie Curie University, Brain and Spine InstituteParis, France; ^6^ National Reference Centre for Unconventional Transmissible AgentsParisFrance; ^7^ Center for Neuropathology and Prion ResearchLudwig Maximilian UniversityMunichGermany; ^8^ Department of Neurology, Institute of Born BungeUniversity of AntwerpAntwerpBelgium; ^9^ Florey Institute of Neuroscience and Mental HealthUniversity of MelbourneMelbourneVictoriaAustralia; ^10^ Department of Molecular Microbiology and ImmunologyNagasaki University Graduate School of Biomedical SciencesNagasakiJapan; ^11^ Prion Laboratory Section, Public Health Agency of CanadaWinnipegManitobaCanada; ^12^ Department of Neurology, University Medical Center and German Center for Neurodegenerative DiseasesUniversity of GöttingenGöttingenGermany; ^13^ Department of MedicineUniversity of MelbourneMelbourneVictoriaAustralia; ^14^ APHP, Pitié‐Salpêtrière HospitalParisFrance; ^15^ Department of Biomedical and Neuromotor Sciences, University of BolognaItaly

## Abstract

Real‐time quaking‐induced conversion (RT‐QuIC) has been proposed as a sensitive diagnostic test for sporadic Creutzfeldt–Jakob disease; however, before this assay can be introduced into clinical practice, its reliability and reproducibility need to be demonstrated. Two international ring trials were undertaken in which a set of 25 cerebrospinal fluid samples were analyzed by a total of 11 different centers using a range of recombinant prion protein substrates and instrumentation. The results show almost complete concordance between the centers and demonstrate that RT‐QuIC is a suitably reliable and robust technique for clinical practice. Ann Neurol 2016;80:160–165

Creutzfeldt–Jakob disease (CJD) belongs to a family of fatal neurodegenerative diseases known as transmissible spongiform encephalopathies (TSEs). TSEs are characterized by the post‐translational conformational change of a normally expressed protein called prion protein (PrP) into a disease‐associated form known as PrP^Sc^. Once formed, PrP^Sc^ can induce PrP to undergo a conformational change and produce more PrP^Sc^ in a self‐propagating manner. The PrP^Sc^ aggregates, becomes protease resistant, and deposits throughout the brain, leading to spongiform change and neuronal loss.

Patients with sporadic CJD (sCJD) present with a rapidly progressing dementia, and death usually occurs within 6 months. Current diagnostic criteria for sCJD rely on clinical features, the results of electroencephalography and magnetic resonance imaging, and the presence of 14‐3‐3 protein in the cerebrospinal fluid (CSF).^1^, ^2^ These tests are not specific for CJD, and none is able to detect all forms of CJD.^3^, ^4^

A new approach to the premortem diagnosis of sCJD has been to exploit the ability of small amounts of CSF PrP^Sc^ to convert native PrP into PrP^Sc^ in a newly described protein aggregation assay known as real‐time quaking‐induced conversion (RT‐QuIC). This technique uses recombinant PrP (rPrP) as a substrate, which is induced to aggregate by the addition of CSF containing PrP^Sc^. Thioflavin T (ThT) in the reaction binds to the aggregated PrP^Sc^, causing a change in the ThT emission spectrum, enabling the reaction to be monitored in real time.^5^, ^6^

CSF RT‐QuIC has been shown to be an accurate diagnostic test for sCJD, with a high degree of sensitivity (85–87%) and specificity (99–100%).^7^, ^8^ An increasing number of laboratories have established RT‐QuIC analysis, and more are interested in doing so. Before RT‐QuIC is fully accepted into clinical practice as a reliable premortem diagnostic test, studies are required to ensure that laboratories performing this assay using different forms of recombinant PrP as a substrate and a variety of instrumentation are producing comparable results.

This study reports the findings of 2 international ring trials that were undertaken over a 2‐year period. The initial ring trial comprised European participants, whereas the second ring trial was wider and included participants from Australia, Canada, and Japan.

## Materials and Methods

### CSF Samples

CSF samples were provided by the National CJD Research & Surveillance Unit, United Kingdom. These samples are stored at &minus;80 °C, and consent was obtained from the next of kin for their use in research (05/MRE00/67). CSF samples were selected on the basis of having appropriate ethical consent and sufficient volume to ensure that each participant had an adequate volume of CSF for analysis. Each set of CSF samples was sent to each laboratory on dry ice and was analyzed blind to the final diagnosis. The performance of the RT‐QuIC assay was evaluated in an interlaboratory ring trial, where identical CSF samples were analyzed by each of the participating laboratories. Using this approach, it may be possible to identify analytical procedures that affect overall assay performance.

### Participants

A total of 11 laboratories participated in the ring trials: Department of Pathology, University of Melbourne, Melbourne, Australia (Aust); Department of Neurology, Antwerp University, Antwerp, Belgium (Bel); Prion Laboratory Section, Public Health Agency of Canada, Winnipeg, Canada (Can); Laboratory Investigations of Alzheimer's Disease and Prion Diseases, Pitié‐Salpêtrière Hospital Group, Paris, France (Fra); Department of Neurology, University Medical Center and German Center for Neurodegenerative Diseases, Göttingen, Germany (Ger‐Goet); Centre for Neuropathology and Prion Research, Ludwig Maximilian University, Munich, Germany (Ger‐Mun); Department of Neurological Sciences, University of Bologna, Bologna, Italy (It‐Bol); Department of Neurodegenerative Disease, Carlo Besta Neurological Institute, Milan, Italy (It‐Mil); Department of Neurological Sciences, National Institute of Health, Rome, Italy (It‐Rome); Department of Molecular Microbiology and Immunology, Nagasaki University, Nagasaki, Japan (Jpn); and National CJD Research & Surveillance Unit, University of Edinburgh, Edinburgh, UK (UK).

### First Ring Trial

Seven laboratories (UK, It‐Rome, It‐Mil, It‐Bol, Fra, Ger‐Mun, Bel) participated in the first ring trial, and each received 10 CSF samples sent on dry ice. Of these, 1 was from a patient with neuropathologically confirmed sCJD, 4 were from patients with probable sCJD, diagnosed according to the World Health Organization criteria, 2 patients improved and the diagnosis of sCJD was excluded on clinical grounds, 1 patient had a steroid‐responsive encephalopathy, 1 had no neuropathological evidence of CJD at postmortem, and 1 had mixed Alzheimer disease and vascular dementia at postmortem. The age of the patients ranged from 48 to 86 years and included 5 females and 5 males. The disease duration of the patients with sCJD ranged from 2 to 12 months, and the CSF samples were taken between 53% and 94% of the disease duration.

### Second Ring Trial

Eleven laboratories (UK, It‐Rome, It‐Mil, It‐Bol, Fra, Ger‐Mun, Ger‐Goet, Jpn, Aust, Can, and Bel) participated in the second ring trial, and each received 15 CSF samples sent on dry ice. One laboratory analyzed the CSF samples twice using a BMG LABTECH (Ortenberg, Germany) Optima and a BMG LABTECH Omega. Of the 15 CSF samples, 5 were from patients with probable sCJD, 3 were from patients with neuropathologically confirmed sCJD, 1 patient improved and the diagnosis of sCJD was excluded on clinical grounds, 1 had seizures, 1 had anti‐immune encephalopathy, 1 had a psychiatric disorder, 1 had Huntington disease, 1 had a non‐CJD dementia, and 1 had normal pressure hydrocephalus. The age of the patients ranged from 55 to 87 years and included 6 females and 9 males. The disease duration of the patients with sCJD ranged from 1 to 26 months and the CSF samples were taken between 57% and 90% of the disease duration.

### Methodology

Each laboratory performed the RT‐QuIC analysis using a standard 10mM phosphate buffer (pH 7.4), 170mM NaCl (total 400mM including phosphate buffer) containing 0.1mg/ml rPrP, 10 μM ThT, and 10 µM ethylenediaminetetraacetic acid tetrasodium salt. However, a range of instrumentations, analytical conditions, and types of rPrP were used (Table 1). Most laboratories used either a BMG LABTECH Optima or a BMG LABTECH Omega, whereas 1 laboratory used a Tecan Infinite F200PRO (Tecan Group, Männedorf, Switzerland). Nine laboratories used hamster full‐length (23–231) rPrP (supplied by Bristol Institute of Blood Sciences, Bristol, UK),^8^, ^9^ 2 used human full‐length (23–231) rPrP (produced in‐house),^7^ and 1 used a hamster–sheep chimeric rPrP (hamster 14–126 residues followed by sheep residues 141‐234, produced according to previously reported conditions).^5^

**Table 1:** Instrumentation, Analytical Conditions, and Source of Recombinant PrP Used by Each of the Participating Centers

Group	Reader	Recombinant PrP	CSF Volume	Shake Conditions	Temp, °C	Criteria for Positive Result
UK Omega	Omega	Ham FL	30 µl	900rpm 90 s shake/30 s rest	42	Mean of 2 highest replicates of 4 > 24,000rfu at 90 h
UK Optima	Optima	Ham FL	30 µl	600rpm 60 s shake/60 s rest	42	Mean of 2 highest replicates of 4 > 10,000rfu at 90 h
Germany (Munich)	Optima	Ham FL	30 µl	600rpm 60 s shake/60 s rest	42	Mean of 2 highest replicates of 4 > 10,000rfu at 90 h
Germany (Göttingen)	Optima	Ham‐Sh chimeric	15 µl	600rpm 60 s shake/60 s rest	42	Mean of 2 highest replicates of 4 > 10,000rfu at 80 h
Italy (Bologna)	Optima	Ham FL	15 µl	600rpm 60 s shake/60 s rest	42	Mean of 2 highest replicates of 4 > 6,000rfu at 90 h
Italy (Milan)	Optima	Ham FL	30 µl	600rpm 60 s shake/60 s rest	42	Mean of 2 highest replicates of 4 > 10,000rfu at 90 h
Italy (Rome)	Omega	Ham FL	30 µl	900rpm 90 s shake/30 s rest	42	Mean of 2 highest replicates of 4 > 32,000rfu at 90 h
France	Omega	Ham FL	30 µl	900rpm 90 s shake/30 s rest	42	Mean of 2 highest replicates of 4 > 34,345rfu at 90 h
Belgium	Omega	Ham FL	20 µl	900rpm 90 s shake/30 s rest	42	Mean of 2 highest replicates of 4 > 20,000rfu at 90 h
Canada	Omega	Ham FL	30 µl	900rpm 90 s shake/30 s rest	42	Mean of 2 highest replicates of 4 > twice baseline reading at 90 h
Japan	Tecan	Hum FL	5 µl	Max 30 s shake/30 s rest	37	At least 2 of 6 replicates > 400rfu at 90 h
Australia	Optima	Hum FL	5 µl	750rpm 30 s shake/30 s rest^a^	37	Mean of 2 highest replicates of 4 > 70% of baseline rfu reading at 90 h

Shaking performed in a Thermomixer Comfort (Eppendorf, Hamburg, Germany) before being read in an Optima instrument.

CSF = cerebrospinal fluid; Ham FL = hamster full‐length PrP (23–231)^8^, ^9^; Ham‐Sh chimeric = hamster residues_14–128_:sheep_141–234_ 5; Hum FL = human full‐length (23–231)—codon 129M^6^; PrP = prion protein; rfu = relative fluorescence units.

## Results

The results of the ring trials are given in Tables 2 and 3. In the first ring trial 6, of 7 laboratories obtained positive RT‐QuIC responses in the CSF samples from all 5 sCJD cases; the remaining laboratory obtained positive RT‐QuIC responses in the CSF of 4 of the 5 sCJD cases. A negative RT‐QuIC result was obtained in the CSF of 1 sCJD case, which had a disease duration of 12 months. This laboratory was using a BMG LABTECH Optima instrument, with full‐length hamster rPrP as substrate and 30 µl of CSF. These conditions were similar to other laboratories in the study that obtained positive results for this particular CSF sample. A limited volume of CSF was sent to each laboratory, and this meant that repeating the analysis of this particular CSF by the individual laboratory was not possible.

**Table 2:** Results for the First Ring Trial with 7 Participating Centers

CSF ID	Diagnosis	Gender (age, yr)	Disease Duration, mo	Timing of LP as % of Disease Duration	Laboratories Reporting Positive RT‐QuIC, No.	Concordance, %
1	Patient improved	M (68)	Still alive	n/a	0/7	100
2	Steroid‐responsive encephalopathy	M (69)	Still alive	n/a	0/7	100
3	Neuropathological evidence of mixed AD and vascular dementia	M (86)	3	92	0/7	100
4	Psychiatric disorder	M (63)	Still alive	n/a	0/7	100
5	Patient improved	F (74)	Still alive	n/a	0/7	100
6	Definite sCJD—codon 129 MM; PrP type: 1	F (75)	3	83	7/7	100
7	Probable sCJD	F (48)	2	77	7/7	100
8	Probable sCJD—codon 129 MM	F (64)	12	94	6/7	83
9	Probable sCJD	F (72)	4	76	7/7	100
10	Probable sCJD—codon 129 VV	M (69)	4	53	7/7	100

AD = Alzheimer disease; CSF = cerebrospinal fluid; F = female; LP = lumbar puncture; M = male; n/a = applicable; PrP = prion protein; RT‐QuIC = real‐time quaking‐induced conversion; sCJD = sporadic Creutzfeldt–Jakob disease.

**Table 3:** Results from the Second Ring Trial with 11 Participating Laboratories

CSF ID	Diagnosis	Gender (age, yr)	Disease Duration, mo	Timing of LP as % of Disease Duration	Laboratories^a^ Reporting Positive RT‐QuIC, No.	Concordance, %
1	Patient improved	F (87)	Still alive	—	0/12	100
2	Seizures	M (56)	—	—	0/12	100
3	Autoimmune encephalitis	F (82)	Still alive	—	0/12	100
4	Psychiatric disorder	M (55)	Still alive	—	0/12	100
5	Huntington disease	F (67)	Still alive	—	0/12	100
6	Mixed vascular and Alzheimer dementia	M (80)	Still alive	—	0/12	100
7	Normal pressure hydrocephalus	M (78)	Still alive	—	0/12	100
8	Definite sCJD	F (63)	6	90	12/12	100
9	Definite sCJD—codon 129 MM; PrP^Sc^ type 1	M (73)	1	71	12/12	100
10	Definite sCJD—codon 129 MM	M (66)	5	57	12/12	100
11	Probable sCJD	M (84)	No data	—	12/12	100
12	Probable sCJD	M (66)	3	83	12/12	100
13	Probable sCJD	M (69)	3	80	12/12	100
14	Probable sCJD	F (65)	26	83	12/12	100
15	Probable sCJD	F (67)	8	88	12/12	100

a The UK laboratory submitted 2 sets of results using 2 sets of instruments: BMG LABTECH Omega and BMG LABTECH Optima.

CSF = cerebrospinal fluid; F = female; LP = lumbar puncture; M = male; PrP^Sc^ = disease‐associated form of prion protein; RT‐QuIC = real‐time quaking‐induced conversion; sCJD = sporadic Creutzfeldt–Jakob disease.

None of the laboratories obtained positive RT‐QuIC responses in any of the 5 CSF samples from patients without sCJD. Four of these laboratories used a BMG LABTECH Optima, and the remaining 3 used a BMG LABTECH Omega. However, all the laboratories used the same full‐length hamster rPrP.

The second ring trial included a larger number of laboratories with a wider geographical distribution and included a wider range of instrumentation and type of rPrP. Despite this, the results showed complete concordance (see Table 3). Of the 11 laboratories that participated in this ring trial, 11 obtained positive RT‐QuIC responses in all 8 CSF samples from sCJD patients. All 11 laboratories obtained a positive RT‐QuIC response in the CSF from the sCJD patient, with a disease duration of 26 months. This CSF sample was taken 21 months after the onset of symptoms. The laboratory that failed to obtain a positive CSF RT‐QuIC response in an sCJD case in the first ring trial correctly identified all sCJD cases in the second ring trial. None of the analytical parameters had been changed by the laboratory in question. Importantly, the laboratory that used a Tecan Infinite shaker with a human rPrP as a substrate also correctly identified all sCJD cases. One laboratory analyzed the ring trial CSF samples using both the BMG LABTECH Optima or a BMG LABTECH Omega and obtained similar results using both instruments despite having different cutoff criteria. None of the laboratories obtained positive RT‐QuIC responses in CSF samples from patients with non‐CJD disorders.

## Discussion

In the first ring trial, 6 of 7 laboratories correctly identified all sCJD cases; however, 1 laboratory obtained a negative CSF RT‐QuIC result from an sCJD patient with a disease duration of 12 months. It has been reported that CSF samples from sCJD patients with longer disease durations may have lower seeding efficiency^10^; however, this effect was not seen in the second ring trial, where the CSF from an sCJD patient with a disease duration of 26 months was identified by RT‐QuIC by all laboratories. All the laboratories that participated in the first ring trial used the same source and type of rPrP and either Optima or Omega BMG LABTECH instrumentation, and demonstrated high levels of accuracy and agreement between laboratories.

The second ring trial took place 18 months later and was expanded to include other European laboratories and participants from Australia, Canada, and Japan. Some of the additional laboratories used alternative types of rPrP and other forms of instrumentation. This enabled a more rigorous assessment of the robustness and transferability of the RT‐QuIC technique.

All 11 laboratories accurately identified the 8 CSF samples from sCJD patients, and none detected a positive RT‐QuIC in any of the non‐CJD cases. The agreement between laboratories using different rPrP as a substrate and different forms of instrumentation is encouraging. The accuracy of the results obtained was identical for each of the rPrP substrates used. From this limited number of CSF samples, we have achieved an overall sensitivity of between 85.7% and 100% and a specificity of 100%. This compares well with a previous intralaboratory study, which had fewer participants and reported a sensitivity of 85% and a specificity of 99%.^10^ The complete concordance between laboratories demonstrates that CSF RT‐QuIC is adaptable to different laboratory instrumentation and different types of rPrP. The high level of accuracy and agreement between laboratories using CSF RT‐QuIC is supportive of this technique being introduced into clinical practice.

## Author Contributions

L.I.M. organized both the ring trials, coanalyzed the data, and cowrote the manuscript; A.P., I.P., S.Su., K.G., S.Sh., B.d.V., S.Sa., K.S., K.C., M.C., G.F., and M.E. performed RT‐QuIC analyses; M.S., I.Z., and P.C. developed RT‐QuIC analysis; L.I.M., F.T., R.A., D.K., S.C., S.H., P.P., M.P., and A.G. participated in the design of the study; A.G. coanalyzed the data and cowrote the manuscript.

## Potential Conflicts of Interest

Nothing to report.
